# Theory of Mind and Reading Comprehension in Deaf and Hard-of-Hearing Signing Children

**DOI:** 10.3389/fpsyg.2016.00854

**Published:** 2016-06-07

**Authors:** Emil Holmer, Mikael Heimann, Mary Rudner

**Affiliations:** ^1^Linnaeus Centre HEAD, Swedish Institute for Disability Research, Department of Behavioural Sciences and Learning, Linköping UniversityLinköping, Sweden; ^2^Infant and Child Lab, Division of Psychology and Swedish Institute for Disability Research, Department of Behavioural Sciences and Learning, Linköping UniversityLinköping, Sweden

**Keywords:** deaf and hard-of-hearing, children, Theory of Mind, sign language, working memory, reading comprehension

## Abstract

Theory of Mind (ToM) is related to reading comprehension in hearing children. In the present study, we investigated progression in ToM in Swedish deaf and hard-of-hearing (DHH) signing children who were learning to read, as well as the association of ToM with reading comprehension. Thirteen children at Swedish state primary schools for DHH children performed a Swedish Sign Language (SSL) version of the Wellman and Liu (2004) ToM scale, along with tests of reading comprehension, SSL comprehension, and working memory. Results indicated that ToM progression did not differ from that reported in previous studies, although ToM development was delayed despite age-appropriate sign language skills. Correlation analysis revealed that ToM was associated with reading comprehension and working memory, but not sign language comprehension. We propose that some factor not investigated in the present study, possibly represented by inference making constrained by working memory capacity, supports both ToM and reading comprehension and may thus explain the results observed in the present study.

## Introduction

Theory of Mind (ToM) is the ability to understand and predict the mental worlds of oneself and others and how they relate to behavior (Frith and Frith, 2012), or, simply, to represent and understand minds. Our understanding of the functional correlates of ToM is still evolving (Carlson et al., 2013); however, one interesting finding is that ToM is associated with reading ability (e.g., Kim, 2015). Early studies assessed ToM using false belief tasks (e.g., Wimmer and Perner, 1983), in which correct solution requires a protagonist’s false belief to be kept in mind. This procedure reflects early conceptualizations of ToM as an all-or-nothing capacity (cf, Baron-Cohen et al., 1985), typically in place at age four (for a meta-analysis, see Wellman et al., 2001). Using a five-point scale, Wellman and Liu (2004) showed that ToM is in fact an ability with a developmental progression, in which representation and understanding of mind emerge in a specific order over time. Their original finding relating to North American children has been replicated in other cultures (e.g., Germany: Henning et al., 2011; China: Wu and Su, 2014; for a review, see Wellman, 2014). According to the five-point scale (Wellman and Liu, 2004), the first stage in ToM development is the ability to understand that the *desires* of oneself and others may not be the same. This ability appears around the age of 2 years in typically developing children. The second stage, typically emerging at the age of three, is the ability to understand that the *beliefs* of oneself and others may differ. The third stage, also emerging at 3 years, is the ability to understand that someone else’s *knowledge* may not be the same as one’s own. The ability to understand *false belief* is the fourth stage in the Wellman and Liu (2004) ToM scale, and this is followed by a fifth stage which involves the ability to understand that displayed and experienced *emotions* may not be the same. The validity of this scale is supported by other work showing that while children have a basic understanding of desires at the age of two, at the age of three, they also start to differentiate between their own beliefs and knowledge and those of others (for reviews, see Carlson et al., 2013; Wellman, 2014). During a similar phase in development, an increase in working memory capacity and executive skills is typically also observed, and the level of these skills seems to constrain development of ToM (Moses and Tahiroglu, 2010; Carlson et al., 2013).

Several disabilities are related to changes in the development of ToM. For example, in children with autism spectrum disorder (ASD), ToM shows atypical developmental progression (Peterson et al., 2005, 2012) which has been attributed to atypical neurobiological development (Lord and Bishop, 2015). In particular, it has been reported that children with ASD have a better ability to understand hidden emotions than false beliefs, possibly because it is easier to form representations of emotions, which are concrete, than of beliefs, which are abstract (Peterson et al., 2005, 2012). From an Australian cultural setting, it has been reported that deaf and hard-of-hearing (DHH) signing children display the same progression in ToM as typically developing hearing children do, but that there might be delays in the age at which different ToM concepts are understood (Peterson et al., 2005, 2012). Such delays have been attributed to socio-cultural factors, including restricted discussion of abstract concepts, including mental states, due to mismatch between the language capabilities of the child and its caregivers (Peterson, 2009; Lederberg et al., 2013; Sundqvist and Heimann, 2014). Mismatch of this nature may arise either because parents underestimate the importance of such speech-based talk or because they lack adequate sign language skills. These situations are common since only about 5% of all DHH children grow up with deaf parents who primarily use sign language themselves (Lederberg et al., 2013). DHH signing children who grow up with hearing parents having restricted knowledge of sign language, typically display delayed ToM development (Peterson, 2009; Lederberg et al., 2013). Other studies have shown that DHH children who have been exposed to a sign language from birth, i.e., DHH native signing children, perform on par with typically developing hearing children on ToM tasks (Lederberg et al., 2013). DHH children with poorer language capabilities are likely to have poorer representations of mental states. According to flexible resource models of working memory, when it is more difficult to form representations it may be harder to process them in working memory (Ma et al., 2014). Thus, delayed development of ToM in DHH children may be due in part to poor language skills and the limitations of working memory. Indeed, associations between ToM and working memory have been reported for DHH children (Meristo and Hjelmquist, 2009). The first purpose of the present study is to determine whether DHH signing children in Sweden follow the typical developmental trajectory in ToM and whether their level of ToM skills is related to working memory and home language.

It is estimated that between 100 and 200 DHH children are born each year in Sweden (Assessing Health Care Interventions, 2006). With the right support, many DHH children can achieve good speech development with technical aids^1^ (Kral and Sharma, 2012), as well as age-appropriate reading skills (Geers et al., 2011; Nakeva von Mentzer et al., 2014; Asker-Árnason et al., 2015). However, there is large variation in speech outcome (Campbell et al., 2014) and some DHH children in Sweden use Swedish Sign Language (SSL; Svartholm, 2010). In order to achieve adequate linguistic development, it is important for these children that SSL is used during both social and learning activities (Svartholm, 2010; Lederberg et al., 2013). Sign languages are natural languages that are used to share thoughts, ideas and beliefs and can be understood at the same linguistic levels as spoken languages but differ from ambient spoken languages in their phonological and syntactic structure (Emmorey, 2002). Thus, sign languages and spoken languages are functionally equivalent. However, sign languages do not have written forms, and DHH children learn to read the written form of the spoken language in the cultural setting in which they grow up, even though their primary language may be signed. Generally, children learn to read by mapping written symbols onto mental representations of speech sounds (Wagner and Torgesen, 1987). When mapping is successful, lexical items are accessed, revealing the meaning of written language (Perfetti and Stafura, 2014). DHH children may lack well-established, speech-based representations. Thus, for DHH children who use sign language, learning to read depends both on the ability to learn a new language system (Perfetti and Sandak, 2000; Trezek et al., 2011), and the ability to utilize sign language skills to understand text (Chamberlain and Mayberry, 2000; Hoffmeister and Caldwell-Harris, 2014).

The bilingual approach to deaf education adopted at Swedish state primary schools for DHH children involves teachers translating written Swedish into SSL and discussing differences between the two languages with the pupils (Svartholm, 2010). Such discussions involve mutual reflection on the child’s thoughts and beliefs about the content of texts. Apart from the intended purpose of supporting reading development, such reflection is likely to promote the ability to differentiate between the thoughts and beliefs of oneself and others that is fundamental to the development of ToM (Wellman, 2014). Furthermore, ToM is likely to influence the establishment of reading skills (Astington and Pelletier, 2005; Blair and Razza, 2007). Indeed, ToM has been shown to explain unique variance in reading comprehension in both typical children (Kim, 2015) and children with ASD (Ricketts et al., 2013). In other words, ToM is likely to be associated with reading comprehension in DHH children. However, to our knowledge, this association has not hitherto been studied. Thus, the second purpose of the present study is to investigate the association of ToM and reading comprehension in DHH children being educated using the bilingual approach. Language comprehension and word reading skills predict reading comprehension in DHH children (Marschark and Wauters, 2008), and they have been estimated to explain around 50% of the variance in reading comprehension in hearing children (Ripoll Salceda et al., 2014). In order to secure variance in reading comprehension ability, while keeping word reading skills in control, we selected participants who had Grade 1 word reading skills. In addition, to rule out general language delays as a factor, we wanted participants to display age-appropriate sign language skills.

In the present study, we investigated ToM in children who are at an early stage of reading development and are attending Swedish state primary schools for DHH children. We predicted typical developmental progression in ToM, although delayed in children with whom caregivers did not primarily use SSL. We also predicted that ToM would be positively related to sign language comprehension and reading comprehension, as well as working memory.

## Materials and Methods

### Participants

Sixteen DHH children (8 boys) with a mean age of 10.1 years (*SD* = 2.1; range 7.3–14.5), attending grades 1–7 in Swedish state primary schools for DHH children, were recruited. Three of the participants had an additional diagnosed medical or developmental disability and were therefore excluded from the study. These individuals performed below the 5th percentile on a test of non-verbal intelligence, i.e., Raven’s Colored Progressive Matrices (Raven and Raven, 1994), indicating possible atypical cognitive functioning. Staff members at the schools selected participants they considered to be at a word reading level corresponding to Grade 1 of hearing children and subsequent testing showed that performance on word reading in the sample did not differ from Grade 1 hearing children (Holmer et al., 2016a). After selection, participants and their parents provided informed consent, attested in writing by the parents. The study was approved by the Regional Ethical Review Board, Linköping, Sweden.

Demographics are presented in **Table 1**. Mean age was 10.2 (*SD* = 2.3). All participants but one performed within the normal range on non-verbal intelligence. This participant scored only one point below (*M* = 25.2, *SD* = 5.88) the normal range and was not excluded since no additional disability was reported. Furthermore, performance on tests of word reading skills of this participant was within ±1*SD* of average performance of Grade 1 hearing children. Two participants had a vision deficit which was corrected. Eleven used technical aids and the mean age at fitting was 3.9 years (*SD* = 2.2). Up-to-date audiological records were not available and since ToM and other cognitive and linguistic skills were the focus of this study, audiological measurements were not made. Seven of the participants were born abroad, one in an expatriate family, and age at which residence in Sweden commenced ranged from 2.2 to 10.6 years (*n* = 5). The age of exposure to SSL was on average 4 years (*SD* = 3; range 0–12). Three participants had been exposed to SSL since birth; two of these participants had parents who where themselves deaf and used SSL. One further participant had parents who primarily used SSL; the rest had parents who spoke a language from Europe, Asia, or Africa, sometimes with the support of signs from SSL when interacting with the participant. The families of three participants partly or fully omitted to provide background data.

**Table 1:** Demographics (*N* = 13).

	*n*
Primary language at home:	
SSL	4
Other	9
Technical aids:	
HA, unilateral	1
HA, bilateral	4
CI, unilateral	1
CI, bilateral	4
HA and CI	1
No aids	2
Educational level of mother:	
University	3
High school	6
Elementary school	1
Not reported	3


*SSL, Swedish Sign Language; HA, Hearing Aid; CI, Cochlear Implant.*

### Measures

#### Theory of Mind

To assess ToM, a Swedish version (Sundqvist et al., 2014a) of the Wellman and Liu’s (2004) five-step ToM scale was adapted for use in SSL (see Procedure below). The Swedish version of the scale was created by translating the original scale in English into Swedish and back-translating into English in consultation with the authors. The scale includes a set of tasks (see **Table 2**) which were administered in an order recommended by Wellman and Liu (2004): diverse desires, knowledge access, content false belief, diverse beliefs, and hidden emotions. The SSL adaptation of the scale differed from standard versions in two ways. First, all names were replaced with their category designator (e.g., “the man”, “the girl”). This choice was made because the particular name was not pertinent to the task and in sign languages all names have to be fingerspelled the first time they are used, probably leading to letter-by-letter representation and increased working memory load, reducing resources for performing the ToM task. The second change was based on recommendations of Peterson et al. (2005); a control question was added to the HE task, i.e., when the child had pointed to the neutral, smiling, or sad face, the child was asked why the protagonist felt that way. In accordance with the standard procedure (Wellman and Liu, 2004), one point was awarded for each of the tasks where both target question and control questions were answered correctly and the total number of tasks solved constituted an index that was used when computing correlations.

**Table 2:** Tasks in the Swedish version of the Theory of Mind scale in ascending order of difficulty (Wellman and Liu, 2004).

Task	Description of task
Diverse desires	The participant has to distinguish between the desires of two different actors (the participant him-/herself and a second party) about the same object. The participant is instructed to choose which of two different snacks (carrot or cookie) he/she prefers, and then to predict which snack the second party who has the opposite preference will choose.
Diverse beliefs	The participant has to distinguish between the different beliefs of two persons (the participant him-/herself and a second party) about the location of an object. The participant states whether he/she believes that the object is located in a garage or a shrubbery, and is then informed that the second party believes the object is located in the other place. After that, without knowing the true whereabouts of the object, the participant has to say where the second party will go and look for the object.
Knowledge access	After learning what is inside a neutral box (a toy dog), the participant has to state whether a person that has never looked inside the box knows what is in it. The participant is also asked whether or not the other person looked inside the box.
Content false belief	Knowing that the true content (a toy pig) of a band aid box is not what is to be expected (band aids), the participant has to imagine what another person who does not know the true content of the box (a person with a false belief) will say is in the box. The participant is also asked whether or not the other person saw what was inside the box.
Hidden emotions	The participant has to demonstrate the ability to understand that a person can express one emotion and experience another. The participant is told a story about a boy who wants his aunt to bring him a toy car; however, the aunt brings the boy a book. Then, the participant has to judge what the boy will feel inside (sad) and display (happy or neutral), by pointing to printed black and white emoticons (sad, happy, and neutral). The participant is also asked to explain why the boy tried not to show that he was sad.


#### Reading Comprehension

A Swedish version of the Woodcock Passage Reading Comprehension (Woodcock, 1998) test was used (i.e., Furnes and Samuelsson, 2009). The participant silently read Swedish sentences and paragraphs of different lengths in which one word was omitted. The placement of the omitted word varied over items. In total, there were 68 passages of text and testing was stopped after a sequence of 6 errors. The first few passages consisted of one short sentence (3 or 4 words) with subsequent passages increasing in difficulty such that the last few included two or three sentences with both principal and subordinate clauses. The participant’s task was to complete the passage by either saying, signing, or writing an appropriate word. The dependent measure was the number of correct answers.

#### Sign Language Comprehension

A version of the British Sign Language Receptive Skills Test (Herman et al., 1999) adapted for SSL was used to assess sign language comprehension. Testing started with a vocabulary check, and then the participant was presented with 40 videos of SSL sentences. For each sentence, the participant judged which picture out of three or four alternatives best matched the meaning of the sentence. The participant was awarded one point for each correct response and the dependent measure was total number of correct answers. Testing was conducted by native SSL users who had been trained to administer the test. For two of the participants, results dating from 10 months prior to testing were available and these participants were not re-tested due to ethical considerations.

#### Working Memory

To assess working memory capacity, a visuo-spatial task called The Clown test (Sundqvist and Rönnberg, 2010; Birberg Thornberg, 2011) was used. The Clown test is based on the Mr Peanut task introduced by Kemps et al. (2000). The participant was presented with a clown figure on a magnetic board, which had a set of colored magnets placed on it in a predefined pattern. After a number of seconds, corresponding to the number of magnets placed on the figure, it was turned away from the participant and the magnets were removed. Then the participant was asked to report the color of the magnets. When a response was given, the participant was asked to replace the magnets in their original configuration or point it out. The number of magnets increased from one to ten across trials, with three trials at each level (30 possible trials in all). On each trial, the original configuration of the magnets had to be correctly specified for a response to be counted as correct, and the participant had to answer correctly on at least two out of three trials with a particular number of magnets to move on to the next level. The participant was awarded one point for each correct trial, and the dependent measure was the total score.

### Procedure

Participants were tested individually at their school by members of staff who were fluent signers and familiar to the participants. In total, there were five test leaders, of whom two deaf native signers administered the test of sign language comprehension. The other three were trained to administer all other tests by the first author. Instructions were available in SSL and in Swedish, and mode of instruction was adapted to the needs of the participant. SSL instructions were provided by the test leader and were based on written instructions following a formalized coding system for rephrasing the Swedish instructions in SSL (Bergman, 2012). This procedure was used to minimize divergences in the instructions different participants were given. Test leaders made sure that the participant understood each task before testing took place, and participants practiced all but the ToM tasks before administration.

For the ToM scale, the rephrasing was done by a licensed sign language interpreter, and was then checked by two native signing teachers of DHH children. In the few instances that disagreement occurred, it was discussed until a consensus was formed. The rest of the instructions were coded by a deaf native SSL user, and checked by three of the test leaders in the study. For practical reasons, even though there was a recommended order, test order was individually adapted and breaks were taken when needed.

### Data Analysis

Descriptive statistics for reading comprehension, sign language comprehension, and working memory are reported elsewhere (Holmer et al., 2016a,b), and here we perform new analyses not previously reported. First, normality assumptions were tested, descriptive statistics were computed and data was visually inspected. Progression on the ToM scale was determined by calculating the proportion of participants who correctly solved each task. The total number of tasks correctly solved by each participant was used as an individual index of ToM ability (cf, Peterson et al., 2005, 2012). We used an independent samples *t*-test to test our prediction that participants with caregivers who mainly used SSL at home would score higher than other participants on the individual index of ToM ability. We then computed correlations (Pearson’s *r*) to investigate our predictions that ToM would be associated with reading comprehension, SSL comprehension and working memory. The parametric approach was applied because Shapiro–Wilk’s test statistics indicated that all variables were normally distributed (*p* > 0.05). A significance level of 0.05 (two-tailed) was applied for all tests. To obtain maximum power, despite low *n*, no correction was made for multiple comparisons and one missing data point on the sign language comprehension test was replaced with group mean when calculating correlations. All statistical computations were conducted using IBM SPSS Statistics (Version 22.0).

## Results

### Descriptive Statistics

**FIGURE 1:**
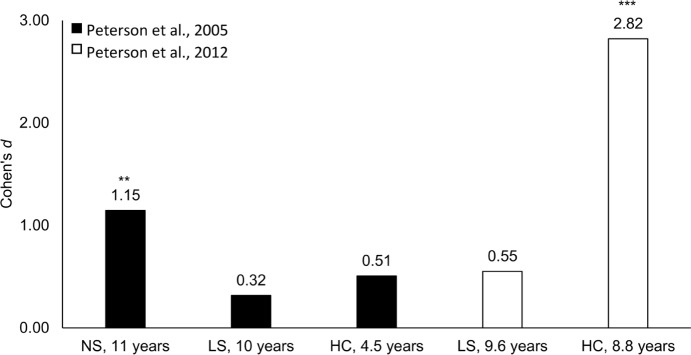
**Effect sizes (Cohen’s *d*) for comparisons of index score on the Theory of Mind (ToM) scale of the present sample with that of selected groups of deaf native signing (NS), deaf late signing (LS), and hearing children (HC) from Peterson et al. (2005, 2012).** The mean age of each comparison group is displayed next to the group label. ^∗∗^One-sample *t*-test, *p* < 0.01. ^∗∗∗^One-sample *t*-test, *p* < 0.001.

In **Table 3**, performance on the ToM scale is shown and compared to published results relating to similar groups. This reveals that the developmental progression of participants in the present study did not differ from that found in previous studies relating to children with typical development (Wellman and Liu, 2004; Peterson et al., 2005, 2012; Henning et al., 2011; Wu and Su, 2014) and DHH signing children (Peterson et al., 2005, 2012). However, it should be observed that there was no difference in the proportion of children who solved the diverse beliefs and knowledge access tasks in the current data set. Furthermore, comparing ToM index of the present sample to that of groups from earlier studies revealed that the participants in the present study were delayed in ToM (see **Table 3** and **Figure 1**). Overall, comparisons indicated small to large between-group effect sizes (Cohen’s *d*, with 0.2 reflecting a small, 0.5 a medium, and 0.8 a large effect size, Cohen, 1992; see **Figure 1**). In particular, performance was worse than the mean score of deaf native signers in Peterson et al. (2005), *t*(12) = 4.23, *p* = 0.001, *d* = 1.15, and that of hearing children in Peterson et al. (2012), *t*(12) = 6.13, *p* < 0.001, *d* = 2.82, despite similar ages across groups. Thus, although developmental progression did not differ from that demonstrated in earlier studies, there was a clear delay in development of ToM.

**Table 3:** Percentage of participants who solved each task on the Theory of Mind (ToM) scale.

	Present study	Wellman and Liu (2004)	Peterson et al. (2005)			Peterson et al. (2012)	
				
	(*N* = 13)	HC (*N* = 75)	NS (*N* = 11)	LS (*N* = 36)	HC (*N* = 62)	LS (*N* = 31)	HC (*N* = 29)
Diverse desires	85	95	100	92	95	94	100
Diverse beliefs	54	84	91	92	85	94	100
Knowledge access	54	73	82	53	82	64	100
Content false belief	46	59	82	33	32	52	100
Hidden emotion	15	32	54	28	19	19	79
ToM index, *M* (*SD*)	2.5 (1.3)	–^a^	4.1 (1.4)	3.0 (1.5)	3.2 (1.3)	3.3 (1.4)	4.8 (0.4)
Age, *M* (*SD*)	10 (2.3)	4.7 (–)	11 (1.8)	10 (2.5)	4.5 (0.6)	9.6 (1.7)	8.8 (1.2)


*Results from previous studies are provided for comparison purposes and ToM index is based on the total number of solved tasks. HC, hearing children; NS, native signing deaf children; LS, late signing deaf children. ^a^Total score on the five-step scale is not reported.*

We have reported (Holmer et al., 2016b) that the performance of the DHH participants in the present study on reading comprehension (*M* = 3.8, *SD* = 1.2) was significantly worse than that of Grade 1 hearing children (*M* = 14, *SD* = 8.8), but that there was no difference in working memory (DHH, *M* = 2.1, *SD* = 0.7; hearing, *M* = 1.8, *SD* = 0.8; a similar pattern from a Swedish context was reported by Rudner et al., 2015).

Mean performance on the SSL comprehension test was 33 (out of a possible 40; *SD* = 5.0, *n* = 12). No norms are available for the SSL version of this test. However, norms are available for the equivalent test in British Sign Language (BSL) for children between the ages of 3 and 12 (Herman and Roy, 2006). One participant in the present study was older than 12 years and performed almost 1 *SD* above the mean according to the BSL norm for 12-year olds. Of the remaining 11 participants, 9 scored within ±2*SD* of the mean according to the BSL norm for their age group and 2 performed even better.

**FIGURE 2:**
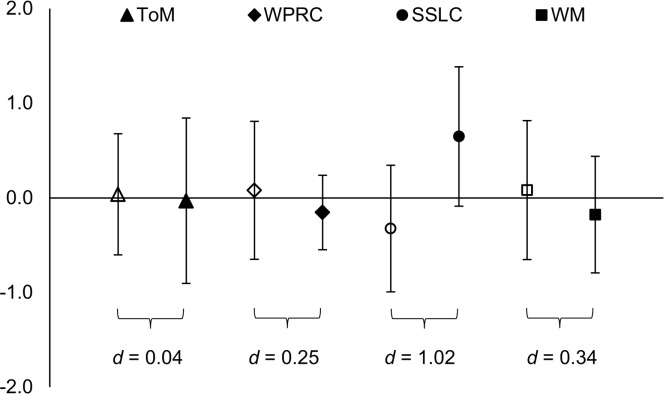
**Comparisons of performance on Theory of Mind (ToM; triangles), Woodcock Passage Reading Comprehension (WPRC; diamonds), Swedish Sign Language Comprehension (SSLC; circles), and Working Memory (WM; squares) between participants with parents who primarily use SSL (*n* = 4; filled) and participants with parents who do not primarily use SSL (*n* = 9; unfilled).** Scores have been standardized, and a value of 0 represents the sample mean performance (*SD* = 1). Bars mark the 95% confidence intervals. Cohen’s *d* indicate the between group effect size (small > 0.20, medium > 0.50, large > 0.80; Cohen, 1992).

Descriptive statistics for all tasks are reported in **Table 4**. Participants with parents who primarily used SSL did not differ from other participants on ToM, *t*(11) = 0.07, *p* = 0.95, *d* = 0.04. In fact, no between-group differences were initially detected on study variables (*t*-test statistics, *p* > 0.05). However, there was a large effect size (*d* > 0.8; Cohen, 1992) on SSL comprehension (see **Figure 2**), suggesting that performance was better among participants with parents who primarily used SSL. When age was entered as a covariate, this difference reached significance, *F*(1, 10) = 5.70, *p* = 0.038, *d* = 1.62.

**Table 4:** Descriptive statistics on study variables for participants with parents who primarily use Swedish Sign Language at home (SSL) and participants with parents who primarily use a spoken language at home (other).

	SSL (*n* = 4)			Other (*n* = 9)^a^		
		
Measure	*M*	*SD*	95% CI	*M*	*SD*	95% CI
Age, years	8.7	1.3	[6.6, 11]	11	2.3	[9.1, 13]
Non-verbal intelligence, raw score	28	5.3	[19, 36]	24	6.1	[20, 29]
Theory of Mind, index	2.5	1.3	[0.5, 4.6]	2.6	1.4	[1.5, 3.7]
WPRC, raw score	3.5	0.6	[2.6, 4.4]	3.9	1.5	[2.8, 5.0]
SSL comprehension, raw score	36	2.1	[33, 40]	31	5.6	[27, 36]
Working memory, raw score	1.9	0.4	[1.2, 2.6]	2.1	0.8	[1.6, 2.7]


*WPRC, Woodcock Passage Reading Comprehension; SSL, Swedish Sign Language; CI, Confidence Interval. ^a^*n* = 8 on SSL comprehension.*

### Correlations

**FIGURE 3:**
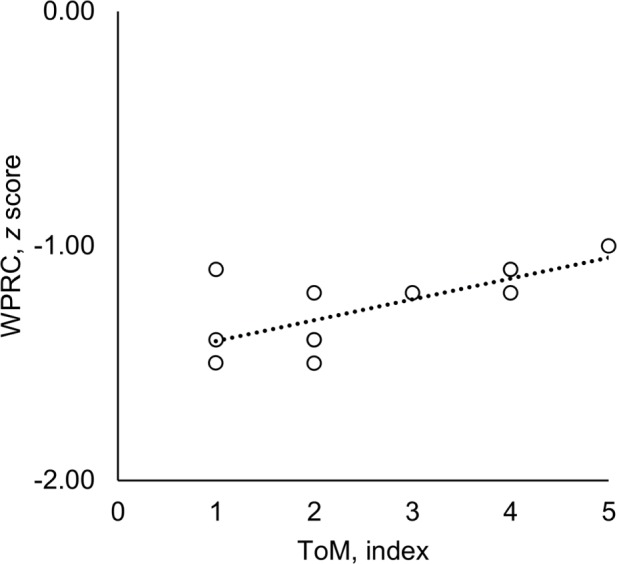
**Association between ToM and *z*-score on Woodcock Passage Reading Comprehension (WPRC; *z*-scores are calculated based on the norm values for Grade 1 hearing children from Furnes and Samuelsson, 2009, i.e., 0 represents mean performance of Grade 1 hearing children, *SD* = 1).** Regression line (dotted) represents the strength of relationship between variables.

Associations between variables are reported in **Table 5**. In accordance with our predictions, index score on the ToM scale was positively associated with reading comprehension, *r*(13) = 0.69, *p* = 0.009 (see **Figure 3**), and working memory, *r*(13) = 0.61, *p* = 0.028. However, contrary to our prediction, no statistically significant association was found between ToM and sign language comprehension, *r*(13) = 0.39, *p* = 0.18. Furthermore, there was no statistically significant correlation between sign language comprehension and reading comprehension, *r*(13) = 0.42, *p* = 0.15, and the association between ToM and reading comprehension was still significant after partialling out the effect of sign language comprehension, *r_p_*(10) = 0.63, *p* = 0.028. Despite the high variability in age of exposure to SSL, there was no association with the ToM index, *r*(10) = 0.11, *p* = 0.66.

**Table 5:** Correlations between study variables.

	1	2	3	4
(1) Theory of Mind		0.69^∗∗^	0.39	0.61^∗^
(2) WPRC			0.42	0.63^∗^
(3) SSL comprehension^a^				0.51
(4) Working memory				


*WPRC, Woodcock Passage Reading Comprehension; SSL, Swedish Sign Language. ^a^One mean imputation. ^∗∗^*p* < 0.01, ^∗^*p* < 0.05.*

## Discussion

In the present study, we investigated ToM in children attending Swedish state primary schools for DHH who use SSL and are at an early stage of their reading development. To achieve this, we used a version of the Wellman and Liu (2004) five-step ToM scale adapted for SSL. The main finding was that the order of progression in ToM development did not differ from that reported for typically developing children (Wellman and Liu, 2004; Henning et al., 2011; Wu and Su, 2014) as well as for DHH signing children (Peterson et al., 2005, 2012) in other cultural settings. However, we did not find that DHH signing children whose parents mainly communicated with them in SSL had more advanced ToM than the other participants, even though their SSL comprehension was better. Furthermore, all participants appeared to be delayed in their ToM, compared to the performance of native signing and typically developing hearing children reported in earlier studies. We did find an association between ToM and both reading comprehension and working memory, in line with our predictions, although not between ToM and SSL comprehension, and the association between ToM and reading comprehension was still significant after controlling for general language skills.

### Progression in Theory of Mind

While early studies regarded ToM as an all-or-nothing capacity (e.g., Baron-Cohen et al., 1985), more recent work has shown that there is a sequence in the development of different aspects of this skill. In particular, Wellman and Liu (2004) showed that there is a typical developmental progression ranging from the understanding of diverse desires and beliefs through knowledge access to understanding of false belief and hidden emotion. Previous work has suggested that DHH children who are not native signers are at risk of delayed ToM development (Peterson, 2009; Lederberg et al., 2013; Sundqvist et al., 2014b).

The results of the present study are in line with previous studies showing typical progression of ToM development in DHH signing children. Importantly, while earlier work has been able to generalize findings of ToM progression in typically developing children from the English-speaking world to cultures with other languages (Henning et al., 2011; Wu and Su, 2014), the present work partially supports generalization of findings of typical ToM progression in DHH signing children from English-speaking cultures to a Swedish setting, and thus lends support to the notion of a progression in ToM during childhood (Wellman, 2014). DHH signing children seem to advance in ToM across a set of developmentally differentiated but psychologically linked achievements in much the same way as typically developing children. However, in contrast to previous studies there was no difference in the percentage of participants who solved the diverse beliefs and knowledge access tasks. Although, our data cannot definitively determine the order of these developmental stages, it does not suggest that it is different in DHH signing children in a Swedish setting from that found in previous studies. It is likely that this phenomenon is related to the process of adapting the scale to a new culture (Wellman, 2014) or random errors. To learn more about the usefulness and psychometric properties of the ToM scale in a Swedish context, future studies should use the scale to investigate ToM development in larger samples of typically developing Swedish children as well as children with diagnoses previously associated with ToM difficulties (e.g., ASD; Lord and Bishop, 2015).

### Delay in Theory of Mind

Although the developmental progression of ToM was not altered in the present study, it was delayed in relation to the ToM performance of DHH native signing and typically developing hearing children of similar age reported in earlier studies (Peterson et al., 2005, 2012). This applied both to participants whose parents primarily used speech and to participants whose parents mainly used SSL, despite the stronger sign language skills of the latter group. It is well established that linguistic environment and establishment of functional language skills influence ToM development in DHH children (Peterson, 2009; Lederberg et al., 2013; Sundqvist and Heimann, 2014). However, another important aspect is the nature of the social interactions in the environment in which development occurs (Reddy, 2008; Wellman, 2014). It has been shown that the degree to which parents adapt their behaviors to the mental world of their infants during social interaction predicts ToM development (Meins et al., 2002; Kirk et al., 2015). Thus, belonging to a sign language rich setting and developing age-appropriate sign language skills may be necessary but not sufficient for typical ToM development in DHH signing children. Investigating parent–child interaction was beyond the scope of the present work, but should be considered in future studies.

ToM performance in the present sample was weaker than that of DHH native signing children in the study by Peterson et al. (2005) but statistical testing did not reveal that it was weaker than that of late signing DHH children reported in previous studies (Peterson et al., 2005, 2012), although effect sizes indicated small to medium mean differences. SSL comprehension for the sample was age appropriate, and thus there was no general language delay that could explain the observed delay in ToM. Furthermore, age of first exposure to SSL was not related to ToM performance. In fact, it is hard to identify any factor taken into account in the present study that can explain the obviously delayed ToM in this group. However, at the same time, we cannot rule out that any of these factors has explanatory value, considering the small and heterogeneous sample as well as the concomitant disproportionately large effect of any confounding variables. In particular, it should be noted that in the group of participants whose parents mainly used SSL, only two were native signers, defined as having at least one deaf signing parent and had been exposed to sign language since birth. Hence, as a group, the present sample may be very similar to late signing groups included in earlier studies, and the lack of association between ToM development and age of SSL exposure on the one hand and general SSL skills on the other should be interpreted with caution.

### Correlations between Theory of Mind, Reading Comprehension, Sign Language Skills and Working Memory

In line with previous work in typically developing children (Kim, 2015) and children with ASD (Ricketts et al., 2013), we observed a positive association between ToM and reading comprehension in the DHH signing participants in the present study. To our knowledge, this is the first time this relationship has been studied in DHH children. In earlier studies, a relationship between ToM and reading comprehension has been discussed in relation to general language skills (Astington and Pelletier, 2005; Ricketts et al., 2013; Kim, 2015), working memory and executive skills as well as inference making (Ricketts et al., 2013; Kim, 2015), and it has been suggested that ToM is a prerequisite for learning socially mediated skills like reading (Frith and Happé, 1994; Ricketts et al., 2013).

#### Lack of an Association between Sign Language Skills and Theory of Mind and Reading Comprehension

Sign language comprehension was not significantly associated either with ToM or with reading comprehension. However, the literature indicates that general language skills and ToM are related in typically developing children (Milligan et al., 2007), DHH signing children (Lederberg et al., 2013), DHH children with technical aids who use speech (Sundqvist and Heimann, 2014), and individuals with dual sensory loss (Frölander et al., 2014). Furthermore, general language skills are related to reading comprehension in typically developing (Ripoll Salceda et al., 2014) as well as DHH (Mayberry et al., 2011) children.

It is possible that the lack of statistically significant associations between sign language skills and ToM as well as reading comprehension is due to low power or heterogeneity of the sample in the present study. However, the lack of association between ToM and sign language comprehension may also be due to the fact that although the sign language test used here provides an estimate of general sign language skills (e.g., Woolfe et al., 2002; Jones et al., 2015), it does not tap onto linguistic aspects of central importance to ToM. For example, it has been suggested that the ability to represent mental states linguistically and to embed propositions under mental verbs, e.g., “He/she thought that …”, is a prerequisite for reasoning about the minds of others (Milligan et al., 2007; de Villiers and de Villiers, 2014), and neither of these aspects was assessed in the present study. Astington and Pelletier (2005) suggested that general language skills explain shared variance between ToM and reading comprehension. However, controlling for general language skills in the present study did not seem to affect the correlation between ToM and reading comprehension. This is in line with findings from a structural equation model (SEM) by Kim (2015), where ToM, vocabulary and grammatical knowledge all explained unique variance in reading comprehension. Ricketts et al. (2013) also reported that ToM predicted unique variance in reading comprehension after controlling for general language skills in children with ASD. Thus, the findings of Kim (2015) and Ricketts et al. (2013) and the correlations between sign language, ToM and reading comprehension in the present study, suggest that a positive relationship between ToM and reading comprehension cannot be completely explained by general language skills.

#### Associations between Working Memory, Theory of Mind and Reading Comprehension

As predicted, working memory capacity was related to both ToM and reading comprehension. In typically developing individuals, working memory is related to comprehension of both texts (Daneman and Merikle, 1996) and minds (Moses and Tahiroglu, 2010; Carlson et al., 2013). Positive relationships between working memory and ToM (Meristo and Hjelmquist, 2009) and between working memory and reading comprehension (Garrison et al., 1997; Daza et al., 2014) have also been reported in DHH individuals. Kim (2015) reported that working memory had a direct relationship to ToM; however, the relationship between working memory and reading comprehension was mediated by vocabulary and ToM.

In the five-step ToM scale (Wellman and Liu, 2004), the working memory demands increase across tasks. In the two most fundamental tasks, diverse desires and diverse beliefs, the participant has to differentiate between their own preference and another person’s preference. Because pictures are provided to support this decision, mental representation is supplemented. However, the more advanced tasks (i.e., Knowledge access, Content false Belief, and Hidden emotion) all rely more on mental representation. To test the constraining influence of working memory capacity on progression in ToM, we suggest adding further tasks to the scale to determine whether individuals who fail to solve the more advanced tasks are able to solve the diverse desires and diverse belief tasks without the support of pictures. If they cannot, this would suggest that working memory capacity constrains performance on the ToM scale.

#### On the Relation between Theory of Mind and Reading Comprehension

Language skills and working memory capacity seem to be important for comprehension of both texts and minds. However, we suggest that neither of these variables on their own, or in combination, can fully explain the set of results of the present study. Kim (2015) and Ricketts et al. (2013) noted that both ToM and reading comprehension involve inference making, and suggested that this ability may link ToM to reading comprehension. Furthermore, Kyle and Cain (2015) showed that both deaf and hearing children who were poor reading comprehenders had poorer inference making skills than hearing controls with good reading comprehension. Since DHH signing children learn to read in a second language, their lack of relevant language-specific background knowledge may make it especially difficult to make appropriate inferences during reading (Hoffmeister and Caldwell-Harris, 2014). Poor ToM has also been suggested to negatively influence skills that rely on socially mediated learning (Frith and Happé, 1994; Scheuffgen et al., 2000; Carlson et al., 2013), such as reading (Ricketts et al., 2013), and it is possible that this is reflected in the relationship between ToM and reading comprehension (cf, Lecce et al., 2011, 2014). However, we tentatively suggest that in addition to working memory and language skills, inference making may play a crucial role in both ToM and reading comprehension and is a plausible mechanism behind the positive correlation between these skills in the present study. Future studies should consider the role of inference making ability, as well as other possible key mechanisms, when further exploring the association between ToM and reading comprehension.

## Conclusion

Children attending Swedish state primary schools for DHH children and who are at an early stage of their reading development, displayed progression in ToM that did not differ from previous studies. However, they had delayed ToM and poor reading comprehension. These skills were positively associated with each other and related to working memory capacity. Our tentative interpretation of this set of results is that some factor not investigated in the present study, possibly represented by inference making constrained by working memory capacity, is involved in constructing a representational model both of minds and of texts.

## Author Contributions

EH, MH, and MR designed the study. EH co-ordinated data collection and performed the statistical analyses. EH prepared the first draft of the article and all authors contributed to the final version.

## Conflict of Interest Statement

The authors declare that the research was conducted in the absence of any commercial or financial relationships that could be construed as a potential conflict of interest.

## Footnotes

1 The term technical aids refers to hearing aids, cochlear implants or a combination of these.
